# A synthetic peptide sensitizes multi-drug resistant *Pseudomonas aeruginosa* to antibiotics for more than two hours and permeabilizes its envelope for twenty hours

**DOI:** 10.1186/s12929-020-00678-3

**Published:** 2020-08-06

**Authors:** Iosu Rázquin-Olazarán, Hawraa Shahrour, Guillermo Martínez-de-Tejada

**Affiliations:** 1 Department of Microbiology and Parasitology, University of Navarra, E-31008 Pamplona, Spain; 2 Laboratory of Microbiology, Department of Life & Earth Sciences, Faculty of Sciences I, Lebanese University, Hadat campus, Beirut, Lebanon; 3 Navarra Institute for Health Research (IdiSNA), Pamplona, Spain

**Keywords:** Antibiotic resistance, *Pseudomonas aeruginosa*. Antimicrobial peptide, Post-antibiotic effect, Permeability

## Abstract

**Background:**

*Pseudomonas aeruginosa* is a Gram-negative pathogen that frequently causes life-threatening infections in immunocompromised patients. We previously showed that subinhibitory concentrations of short synthetic peptides permeabilize *P. aeruginosa* and enhance the lethal action of co-administered antibiotics.

**Methods:**

Long-term permeabilization caused by exposure of multidrug-resistant *P. aeruginosa* strains to peptide P4–9 was investigated by measuring the uptake of several antibiotics and fluorescent probes and by using confocal imaging and atomic force microscopy.

**Results:**

We demonstrated that P4–9, a 13-amino acid peptide, induces a growth delay (i.e. post-antibiotic effect) of 1.3 h on a multidrug-resistant *P. aeruginosa* clinical isolate*.* Remarkably, when an independently P4–9-treated culture was allowed to grow in the absence of the peptide, cells remained sensitive to subinhibitory concentrations of antibiotics such as ceftazidime, fosfomycin and erythromycin for at least 2 h. We designated this persistent sensitization to antibiotics occurring in the absence of the sensitizing agent as Post-Antibiotic Effect associated Permeabilization (PAEP). Using atomic force microscopy, we showed that exposure to P4–9 induces profound alterations on the bacterial surface and that treated cells need at least 2 h of growth to repair those lesions. During PAEP, *P. aeruginosa* mutants overexpressing either the efflux pump MexAB-OprM system or the AmpC β-lactamase were rendered sensitive to antibiotics that are known substrates of those mechanisms of resistance. Finally, we showed for the first time that the descendants of bacteria surviving exposure to a membrane disturbing peptide retain a significant level of permeability to hydrophobic compounds, including propidium iodide, even after 20 h of growth in the absence of the peptide.

**Conclusions:**

The phenomenon of long-term sensitization to antibiotics shown here may have important therapeutic implications for a combined peptide-antibiotic treatment because the peptide would not need to be present to exert its antibiotic enhancing activity as long as the target organism retains sensitization to the antibiotic.

## Background

Due to the progressive shortage of available therapeutic options, the control of infections due to multidrug-resistant (MDR) microorganisms is one of the greatest challenges of modern medicine [1, 2]. Antimicrobial therapies are even scarcer when the causative organism is a Gram-negative MDR bacterial pathogen, such as *P. aeruginosa* [3, 4]*.* This situation has prompted an intense search for therapies based on alternative agents, such as antimicrobial peptides (AMPs) that showed a potential ability to overcome bacterial resistance mechanisms [5, 6]. A widely used last resort antimicrobial peptide is colistin, which often is the only compound that retains activity on MDR strains of *P. aeruginosa* [7, 8]. AMPs are important components of the first line of host defense and constitute a mechanism of innate immunity that has been conserved throughout evolution [9, 10].

Our research group focuses on the use of AMPs as enhancers of the activity of conventional antibiotics against resistant bacterial strains. This property –shared by many AMPs- is based on the ability of these agents to bind to specific molecules of the bacterial envelope and to permeabilize the cell membrane [11–13]. Their permeabilizing activity enables AMPs to sensitize bacteria to co-administered antibiotics, a phenomenon that can be exploited to restore the sensitivity of MDR organisms to antibiotics that had no activity in the absence of the enhancer [8, 14]. Using a rational design method, our group has shown that the permeabilizing ability of peptides based on the LPS binding domain of human lactoferricin (LF11 peptide) can be efficiently improved [15]. The lead compound obtained in this optimization process sensitized *P. aeruginosa* to erythromycin both in vitro and in vivo [15].

The capacity of a given drug to induce post-antibiotic effect (PAE) significantly increases its therapeutic potential. PAE is defined as the suppression of bacterial growth that persists after short exposure of organisms to antimicrobials [16, 17]. Induction of PAE by a drug has important clinical implications, since the desired therapeutic effects can be achieved with fewer doses of the compound. This may result in a more convenient dosage regimen for the patient and a potential reduction of both treatment toxicity and healthcare costs [18, 19].

Except for colistin [20–23] and proline-rich peptides [24], few reports studied PAE of AMPs in detail, although at least melittin [25] and lactoferricin [26] have been reported to induce PAE. Since the AMPs used in the present study are based on the structure–activity development of the peptide LF11 originating from human lactoferricin and were designed to be co-administered with antibiotics, we hypothesized that they could induce PAE and that this phenomenon could influence bacterial susceptibility to antibiotics.

## Methods

### Bacterial strains and culture conditions

Some relevant characteristics of the bacterial strains used in this study are summarized in Table 1. Strains were grown at 37 °C in Trypticase Soy Broth (TSB; BioMerieux, Mercy l’Etoile, France) supplemented with 16 g/L of agar (TSA; Pronadisa, Alcobendas-Madrid, Spain) when needed. For susceptibility testing and post-antibiotic effect assays, cation-adjusted Mueller-Hinton (MH) medium (Difco Laboratories, Detroit, MI) was used.

**Table 1:** Origin and relevant features of bacterial strains used in this work

Strain	Relevant features	Source or reference
*Pseudomonas aeruginosa*		
PAO1	Wild-type	CECT^a^ 4122
PAO1-GFP	PAO1 (pMF230); PAO1 derivative constitutively expressing GFP	[27]
4158–02 Ps4	Multidrug resistant clinical isolate	[13]
LC1–6	PAO1 derivative overexpressing MexAB-OprM efflux pump	[28]
PAΔAD	PAO1 *ampD*; PAO1 derivative overexpressing AmpC	[29]
PAΔDDh2Dh3	PAO1 *ampD-ampDh2-ampDh3;* PAO1 derivative constitutively overexpressing AmpC	[29]

^a^. Spanish type culture collection

### Peptides and chemicals

Peptides P4–8 (PFWRIRIRRWIRR-NH_2_), P4–9 (PFWRIRIRRWWRR-NH_2_), and P4–18 (FWIRIWRIWRR-NH_2_) were purchased from PolyPeptide Laboratories (Strasbourg, France). Synthesis was carried out using 9-Fluorenylmethyloxycarbonyl (FMOC) solid-phase chemistry. Peptides were purified by RP-HPLC (96% of purity, at least), and their amino acid composition and sequence were confirmed by HPLC and mass spectrometry analysis, respectively. Unless otherwise specified, all the antibiotics and chemical compounds were purchased from Sigma-Aldrich (St. Louis, MO). Antibiotic solutions were prepared and stored according to manufacturer’s recommendations.

### Susceptibility assays and synergy testing

The qualitative pattern of antibiotic susceptibility (antibiogram) of the clinical isolate *P. aeruginosa* Ps4 was obtained using an automated Vitek II system (bioMérieux) equipped with the AST-NO22 card (Table S1). Results were interpreted according to European Committee on Antimicrobial Susceptibility Testing (EUCAST) breakpoints. The minimum inhibitory concentration (MIC) of antibiotics and peptides was determined in cation adjusted MH medium by the microdilution technique following CLSI guidelines [30], as previously described [15]. MIC was defined as the minimum concentration of antimicrobial necessary to inhibit growth after 18 h and was determined visually. Minimum bactericidal concentration (MBC) was determined by plating aliquots from wells without growth onto MH agar plates. MBC was defined as the lowest concentration of the peptide causing a 99.9% loss of viability with respect to the CFU/mL inoculated.

Synergy testing was assessed by the checkerboard method in cation adjusted MH medium, as previously described [15]. Combinations were classified as synergistic if their fractional inhibitory concentration (FIC) index was equal or lower than 0.5.

MIC was also measured in an automated optical analyzer Bioscreen C (Labsystems Laboratories, Helsinki, Finland) in cation adjusted MH medium, as detailed elsewhere [31]. Bioscreen C monitors at regular intervals the turbidity of bacterial cultures growing in microplate wells. Susceptibility assays were carried out in 100-well Bioscreen plates at 37 °C for 24 h with continuous shaking and the absorbance of the cultures was measured every 15 min at 600 nm. The antibiotic concentration causing no delay in bacterial growth was chosen for subsequent Post-Antibiotic-Effect-associated Permeabilization (PAEP) assays (see below).

### Post-antibiotic effect (PAE)

**Fig. 1:**
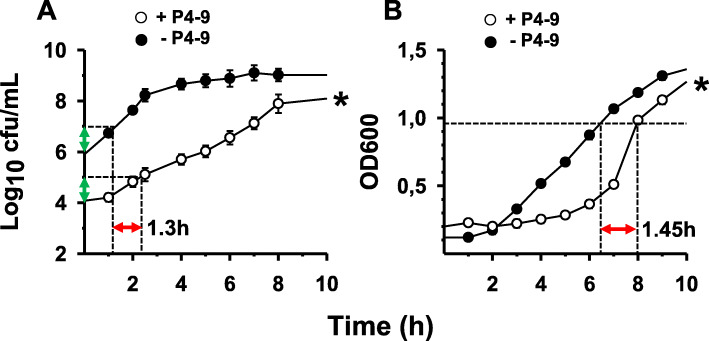
Post-antibiotic effect induced by peptide P4–9 in *P. aeruginosa* Ps4. Growth curves of cultures not previously exposed (solid circles) or preexposed to P4–9 (open circles) at twice the peptide MIC for 1 h. Time 0 corresponds to the beginning of growth monitoring immediately after peptide removal and cell resuspensión in fresh MH medium. **a**, conventional viable count based technique: the 10-fold increments in cell number used to calculate PAE (red arrow) are indicated with green arrows; **b**, turbidimetry-based system (Bioscreen C): the red arrow indicates the value of PAE, which corresponds to the delay undergone by the treated culture with respect to the untreated control in reaching an OD value half of the final OD (i.e. 1.9/2 = 0.95; note that final OD value after 15 h of incubation is not shown). Error bars overlap with symbols in most of the time-points of panel **b**. For a detailed explanation on PAE calculation, see the Methods section. Assays were repeated three times independently and each experiment was performed in triplicate. Data were analysed by Mann–Whitney’s U test and statistical differences in growth kinetics between the treated and untreated cultures were significant (∗; *P* = 0.002, both for panel **a** and **b**)

PAE was defined as the suppression of bacterial growth that persists after short exposure of organisms to antimicrobials [32]. PAE of peptides was determined by two different methods, namely viable counts and turbidimetry [18] with both sharing the initial steps. Inoculum from a fresh culture of *P. aeruginosa* Ps4 was grown in cation adjusted MH medium to mid-log phase (O.D of 0.2–0.3 at 580 nm, corresponding to 10^8^ CFU/mL, approximately). This culture was diluted 10 times in the same medium and 1 mL of this suspension was incubated with the antimicrobial agent at a final concentration of twice the MIC of the compound on Ps4 (i.e. a final concentration of 64 μg/mL for P4–9) for 1 h at 37 °C. To eliminate the antimicrobial, suspensions were centrifuged at 1200 x *g* for 10 min and then the upper portion of the supernatant (90% of the total, approximately) was removed and the volume adjusted to 1 mL with fresh MH medium prewarmed at 37 °C. This washing step was repeated three times. As a control for the calculation of PAE, a duplicate suspension not treated with the antimicrobial was subjected to all the previous steps. After washing, tubes containing 1 mL of bacterial suspension were processed as follows:

I. Viable count-based method: tubes were incubated at 37 °C in an orbital shaker for 24 h and samples were taken at different times (0, 1, 2, 3, 4, 5, 6, 7, 8 and 24 h), and plated onto TSA agar for viable counts. Plates were incubated for 24 h at 37 °C and PAE was calculated according to the following equation [33]: PAE = *T- C*, where *T* and *C* are the time in hours required for the counts in CFU in the treated and untreated cultures, respectively, to increase 1 log_10_ (10-fold) the count observed immediately after drug removal -for the treated culture- or the equivalent time-point for the untreated control (see Fig. 1a for a graphical explanation).
II. Turbidimetry: a 300 μL aliquot from the suspension was dispensed in triplicate into a Bioscreen Honeycomb plate and incubated as detailed above for the Bioscreen-based MIC assay. PAE was calculated according to the following formula: PAE = *T*_50_-*C*_50_, where *T*_50_ and *C*_50_ are the time in hours required for the drug treated and untreated cultures, respectively, to reach a value of optical density corresponding to 50% of the final absorbance reached by an untreated control ( [34]; see Fig. 1b for a graphical explanation). Normally, cultures reached the maximum level of absorbance 15 h after the beginning of incubation and the final value of absorbance was very similar for treated and untreated cultures (1.8–1.9, approximately). To minimize the differences in inoculum size between the treated and untreated cultures -due to the bactericidal activity of the drug-, the untreated control was chosen so that it matched the CFU/mL of the treated culture at the time of growth resumption. This made it necessary to grow several untreated cultures having progressively lower inoculum size in parallel with the treated culture. The CFU/mL of the treated culture at the time of growth resumption was determined by viable counts and then PAE was calculated choosing the data (previously recorded) from the control with inoculum size comparable to that of the test culture.

For every combination of strain and antibiotic, assays I and II were repeated three times independently and each experiment was performed in triplicate. Data were analysed by using the Mann–Whitney’s U test.

### Cytotoxicity

P4–9 toxicity on a human fibroblast cell line was evaluated by the MTT (3-(4,5-dimethylthiazol-2-yl)-2,5-diphenyltetrazolium bromide) test, as previously described [35] using melittin as cytotoxicity control. Hemolytic activity of P4–9 was determined in blood from human volunteers diluted 1:10 in PBS. After 30 min of incubation at 37 °C, absorbance of supernatants was measured at 540 nm and compared to that of erythrocytes treated with Triton X-100 (i.e. 100% of lysis). All the assays were carried out three times independently.

### Post-antibiotic effect associated permeabilization (PAEP)

**Fig. 2:**
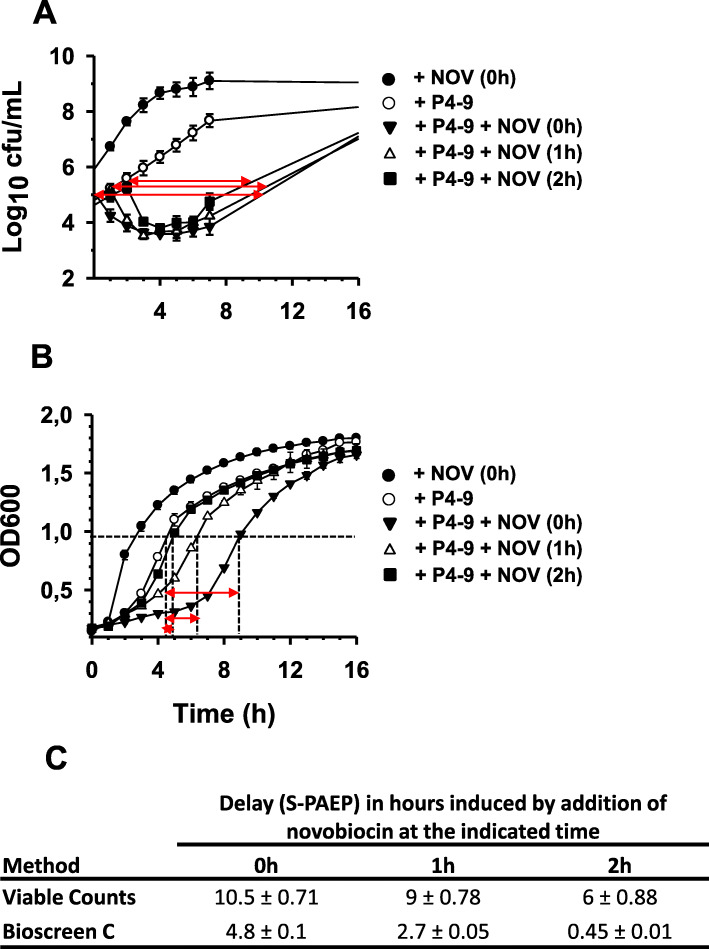
Identification of Post-antibiotic Effect associated Permeabilization (PAEP). Growth curves of Ps4 cultures not previously exposed (solid circles) or preexposed to peptide P4–9 at twice the peptide MIC for 1 h and then treated with a sub-MIC concentration of novobiocin. Time 0 corresponds to the beginning of growth monitoring immediately after peptide removal and cell resuspension in fresh MH medium. At times 0, 1 and 2 h after growth resumption, independent cultures pre-treated with the peptide were exposed to novobiocin at 1/8 x MIC and growth kinetics was monitored by viable counts (**a**) or by using the Bioscreen C system (**b**). Table in panel **c** shows the delay in hours (S-PAEP) caused by each treatment as indicated graphically by red arrows. As control, a P4–9 treated culture was left unexposed to novobiocin (open circles). PAEP was calculated as the average of two independent experiments performed in triplicate. Data were analysed by Kruskal–Wallis test followed by Mann–Whitney’s U test and statistical differences in growth kinetics between cultures exposed to novobiocin at different times (i.e. 0 h vs. 1 h; 0 h vs. 2 h; 1 h vs. 2 h) were significant (∗; P = 0.002). Error bars overlap with symbols in most of the plots. For a detailed explanation on PAEP and S-PAEP calculation, see the Methods section

Post-Antibiotic-Effect-associated Permeabilization (PAEP) was defined as the sensitization of a culture to subinhibitory concentrations of an antimicrobial agent caused by a previous treatment with a PAE-inducing agent that was no longer present. The magnitude of PAEP was quantified by measuring the time it took the antibiotic-treated culture to recover the size it had before antibiotic exposure and this parameter was designated as “Score of Post-Antibiotic Effect-associated Permeabilization (S-PAEP)”. For the determination of S-PAEP, the initial steps of the PAE protocol were strictly applied and then one of the two methods detailed above for PAE calculation was followed with slight modifications. After drug removal by washing (see above), tubes containing 1 mL of drug-treated and untreated bacterial suspensions in MH medium were incubated at 37 °C with selected antibiotics at subinhibitory concentrations. Antibiotic addition was progressively delayed (0, 1, 2, 3 h) with respect to the beginning of incubation. For the viable count-based technique, S-PAEP was calculated by applying the following formula: S-PAEP (h) = *T – t*, where *t* was the time at which the antibiotic was added to the culture and T was the time at which the culture reached the same inoculum that had at the moment of antibiotic addition (see Fig. 2a for a graphical explanation).

When the Bioscreen-based technique was used, the method proposed by Lowdin and collaborators for PAE calculation ([34]; see above) was modified and applied to the determination of S-PAEP using the following formula:

S-PAEP (h) = *TT*_50_ - *TC*_50_ where *TT*_50_ is the time required for the antibiotic-treated culture (previously treated with the peptide or drug under study) to reach a value of absorbance corresponding to half the final absorbance of the culture not treated with antibiotic but previously exposed to the drug, whereas *TC*_50_ is the time required for the culture treated only with the drug (i.e. not treated with antibiotic) to reach the same value of absorbance (see Fig. 2b for a graphical explanation). Potential interferences due to differences in inoculum size between the cultures treated and untreated with the firstly applied drug were minimized by following the method described above for PAE determination.

For every combination of strain and antibiotic, PAEP was calculated as the average of two independent experiments performed in triplicate and data were analysed by Kruskal–Wallis test followed by Mann–Whitney’s U test.

### Fluorescence intensity measurements with NPN

In independent experiments, PAEP was assessed by measuring the amount of the fluorescent probe N-Phenyl-1-naphthylamine (NPN; Sigma) taken up by P4–9 treated cells that, after peptide removal, were allowed to grow in fresh MH medium for increasingly longer time intervals. The initial steps of these assays (i.e. bacterial growth, exposure of cells to P4–9, peptide elimination and growth in the absence of peptide) were performed exactly as described in the previous section (PAE protocol). At different times after peptide removal, cells were harvested by centrifugation (13,000 x *g*; 2 min) and suspended at an OD600 of 0.6 in PBS, pH 7.2. Then, aliquots of 200 μL of this suspension were added to the wells of a 96-well black polystyrene flat bottom microplate (Corning; Sigma-Aldrich) and 4 μL of a 5 mM solution of NPN in acetone was dispensed into each well (i.e. final concentration of NPN was 100 μM). NPN-cell mixtures were homogenized by repeatedly pipetting up and down and immediately afterward fluorescence was quantified in a BMG Labtechnologies FLUOstar Galaxy (BMG LABTECH GmbH, Germany) fluorimeter using an excitation and emission wavelength of 340 and 410 nm, respectively, and a window width of 2.5 nm. Final concentration of acetone (0.3 mM) was at least 2 orders of magnitude below the toxicity threshold of this organic solvent for Gram-negative bacteria [36]. Results were the average of three independent experiments performed in quadruplicate and data were analyzed using two-sample t-test.

### Confocal microscopy

Ps4 was grown and treated with P4–9 as described above (PAE protocol) and then peptide was removed by centrifugation (3 times at 1200 x *g*) and cells were incubated in MH medium in the absence of the peptide for 20 h at 37 °C. In parallel, a culture not exposed to P4–9 was subjected to identical steps. Peptide-treated and untreated cells were washed in PBS and stained with propidium iodide at a final concentration of 1.5 μM. Cells were visualized using a confocal laser scanning microscope (Cell Observer Z1 microscope, Zeiss, Oberkochen, Germany) with a 63x objective. Image acquisition was carried out with the Zeiss software package, and images were processed with ImageJ (ImageJ/Fiji 1.46, National Institution of Health, USA).

### Atomic force microscopy

Cells were prepared for AFM microscopy as described above (PAE protocol), except that, after exposure to the antimicrobial agent, rinsing was performed with 20 mM Hepes pH 7.2 instead of MH medium. Specimens (20 μL of the washed suspension) were placed on glass coverslips (24 × 24 mm; Menzel-Gläser; Braunschweig, Germany) and allowed to dry at room temperature. Coverslips had been previously subjected to an extensive washing process consisting of water-bath sonication for 2 min in a 2% solution of PCC detergent (Thermo Scientific, Rockford, IL) in ultrapure water, followed by rinsing with ultrapure water and a final wash in methanol. To increase adhesion of bacteria to glass [37], coverslips were placed into a 0.01% poly-L-lysine solution in ultrapure water for 5 min and then allowed to dry at room temperature. Images were obtained at room temperature using an AFM microscope (JPK Instruments, Berlin, Germany) equipped with a silicon-nitride cantilever (0.01 to 0.025 Ω / cm, L = 125 μm, W = 35 μm, T = 4.5 μm) operating at 200–400 kHz and 25–75 N/m. For two-dimensional imaging, 10 to 20 cells were visualized at low magnification. If the appearance of all the cells in the field was similar, representative cells were chosen for high magnification imaging and for cross-sectional analysis.

### Conventional fluorescence microscopy

For these assays, a derivative of *P. aeruginosa* PAO1 expressing the green fluorescent protein, the PAO1-GFP strain, was used (Table 1). Preparation of bacterial inoculum, treatment with antimicrobial agents (P4–9 or PMB) and drug removal were performed as described above (PAE protocol). MICs of P4–9 and PMB on PAO1-GFP were 32 μg/mL and 1 μg/mL, respectively. Washed cells were fixed by resuspension in 1 mL of a 4% (w/v) paraformaldehyde (Merck, Madrid, Spain) solution in saline for 10 min at room temperature. After two washes with saline, cells were resuspended in a saline solution containing propidium iodide at a final concentration of 1.5 μM. To remove fluorochrome excess cells were washed twice with saline and 20 μL of suspension was placed on a slide and allowed to dry at room temperature. Visualization was performed using a fluorescence microscope (Nikon Eclipse TS100; Nikon Instruments Inc., Tokyo, Japan) equipped with FITC and TRITC filters to enhance the green and red areas, respectively. Images were digitally overlapped using the computer program Isis FISH Imaging System (MetaSystems, GmbH, Altlussheim Germany).

## Results

### Quantification of the post-antibiotic effect (PAE) of peptide P4–9 on *Pseudomonas aeruginosa* 4158–02 Ps4

The strain used throughout the present study, *P. aeruginosa* 4158–02 Ps4 (henceforth referred to as Ps4), is a multidrug-resistant clinical isolate originally described by Sánchez-Gómez and collaborators ([13]; see antibiogram in Table S1). According to the majority of authors, an antimicrobial agent causes PAE when -immediately after its removal- it brings about a growth delay of at least 0.5 h on a susceptible culture. To study whether our synthetic peptides possessed this property, a log-phase culture of *P. aeruginosa* Ps4 was exposed to a concentration twice the minimum inhibitory concentration (MIC) of the corresponding peptide for 1 h. After peptide removal by thorough washing, surviving cells were allowed to grow in the absence of peptide and growth kinetics was monitored by viable count. For these assays, we selected the three compounds with the lowest MIC from our peptide library, the peptides P4–8, P4–9 and P4–18. Whereas PAE of P4–8 and P4–18 was not significant (data not shown), P4–9 caused a PAE of approximately 1.3 h (Fig. 1a).

PAEs were also determined by an automated turbidimetry-based system (Bioscreen C), which monitors bacterial growth kinetics and is much less laborious than the viable count based method. As shown in Fig. 1b, PAE of P4–9 calculated by the automated method was slightly higher (1.45 h), than that obtained by the conventional technique. On the other hand, the peptide concentration used in these assays (2 x MIC = 64 μg/mL) was still far from being cytotoxic to human cells, as analyzed by the MTT test (half maximal inhibitory concentration (IC_50_) = 164 μg/mL) or by its ability to lyse human red blood cells (half maximal hemolytic concentration (HC_50_)= > 200 μg/mL). In comparison, the bee venom peptide melittin exhibited an IC_50_ of 5.48 μg/mL, and a HC_50_ of 4.92 μg/mL under the same conditions.

### Identification of post-antibiotic effect-associated permeabilization (PAEP)

Since the mechanism of action of most antimicrobial peptides involves disturbance of the cell envelope, we hypothesized that cells that survived treatment with P4–9 could have structural alterations at that level. An indirect evidence of the existence of such abnormalities could be the demonstration that the PAE was associated in those cells with an increase in permeability to antibiotics that are repelled by a functional cell envelope. To study this hypothesis, we treated Ps4 with P4–9 as above and, after removal of the peptide by thorough washing, cells suspended in fresh culture medium were exposed to subinhibitory concentrations of novobiocin, an antibiotic to which *P. aeruginosa* is highly resistant. For these assays, we used a concentration of novobiocin (1/8 the MIC) that in preliminary experiments showed a null inhibitory effect on Ps4 growth.

In the first assay, the antibiotic was added to the P4–9 treated culture immediately after peptide removal and cell resuspension in fresh medium. In this experiment (Fig. 2a), the addition of novobiocin had an instantaneous lethal effect on the P4–9 treated culture, whereas a duplicate culture that was left untreated with the peptide was insensitive to novobiocin. In further experiments, P4–9 treated cells were washed thoroughly, suspended in fresh culture medium and allowed to grow undisturbed for 1 h, 2 h or 3 h before the addition of the antibiotic. Interestingly, cells retained the sensitivity to novobiocin even after 2 h of growth in the absence of the peptide. To our knowledge, this is the first demonstration that sensitization to an antibiotic can persist for several hours of growth in the absence of the sensitizing agent. We coined the term “Post-Antibiotic Effect-associated Permeabilization” (PAEP) to define the sensitization to subinhibitory concentrations of an antimicrobial agent caused by a previous treatment with a PAE-inducing agent that is no longer present.

PAEP was a transient phenomenon since sensitivity to novobiocin was not detectable in cells grown for 3 h in the absence of the peptide (data not shown). Therefore, PAEP induced by P4–9 lasted more than 2 h but less than 3 h. In addition, sensitivity to novobiocin during the PAEP period progressively diminished as the interval between peptide removal and antibiotic addition increased (Fig. 2). Precisely, to quantify the magnitude of sensitization that a culture undergoing PAEP shows to sub-MIC antibiotic concentrations we coined the term “Score of Post-Antibiotic Effect associated Permeabilization” (S-PAEP). This parameter measures the time that an antibiotic-treated culture needs to recover the cell number it had before exposure to the antibiotic during the PAEP period. As shown in Fig. 2a and c, the S-PAEP of novobiocin (or S_nov_-PAEP) added at 0, 1, and 2 h of growth in the absence of the peptide was approximately 10.5 h, 9 h and 6 h, respectively. In contrast, lysozyme caused no reduction of viability on Ps4 when this hydrolytic enzyme was added during PAEP period (data not shown).

Furthermore, we investigated whether these observations could be reproduced using the Bioscreen C system. Although less prominent than in the viable count based method, PAEP was also detectable by the turbidimetric method (Fig. 2b) and manifested itself by a growth delay in the three cultures treated with novobiocin relative to the untreated control (all four pretreated with P4–9). Consistent with our results with the conventional technique, the magnitude of this delay (S_nov_-PAEP) inversely correlated with the length of the interval between the beginning of growth and the addition of novobiocin (4.8 h, 2.7 h and 0.45 h at times 0, 1 and 2 h, respectively; Fig. 2b, Fig. 2c and Table 2) and it was undetectable in cultures exposed to the antibiotic after 3 h of growth (data not shown). Since both methods rendered comparable information, because of its technical advantages, the turbidimetry based system was selected for subsequent experiments.

**Table 2:** Post-Antibiotic Effect (PAE) and Score of Post-Antibiotic Effect-associated Permeabilization (S-PAEP) induced by P4–9

Strain	PAE^**a**^(h)	PAEP^**b**^ (h)	Antibiotic (MIC)^**c**^	Final concentration in μg/mL (times x MIC)	S-PAEP^d^ (h)		
					0	1	2
Ps4	1.45 + 0.02	2–3	NOV (512)	64 (1/8)	4.8 + 0.1	2.7 + 0.05	0.45 + 0.01
		2–3	FOS (512)	64 (1/8)	2.27 + 0.05	2.12 + 0.04	1.60 + 0.03
		2–3	RIF (16)	2 (1/8)	0.6 + 0.01	0.6 + 0.01	0.25 + 0.01
		2–3	ERY (128)	16 (1/8)	1.04 + 0.02	0.82 + 0.02	0.78 + 0.02
		2–3	CAZ (64)	16 (1/4)	14.24 + 0.3	14.24 + 0.3	1.56 + 0.03
		2–3	CAZ (64)	4 (1/16)	2.0 + 0.04	1.25 + 0.03	0.1 + 0.01
		2–3	CEF (1024)	512 (1/2)	0.8 + 0.02	0.7 + 0.01	0.7 + 0.01
		n.f^e^	CIP (2)	0.25 (1/8)	0.7 + 0.01	< 0.25	< 0.25
PAO1	1.9 + 0.03	2–3	NOV (1024)	64 (1/16)	7.81 + 0.16	2.58 + 0.05	0.87 + 0.02
LC1–6	0.75 + 0.04	2–3	NOV (8192)	512 (1/16)	7.62 + 0.15	4.29 + 0.1	0.87 + 0.02
PAΔADD	1.2 + 0.026	2–3	CAZ (64)	16 (1/4)	4.27 + 0.1	3.42 + 0.07	3.32 + 0.07
h2Dh3							

^a^ Post-antibiotic effect assessed by turdidimetry (Bioscreen). PAEs y S-PAEPs values are the average of three and two independent experiments, respectively, each of them performed in triplicate. Differences were analyzed by Kruskal–Wallis test followed by Mann–Whitney’s U test

^b^ Duration of Post-Antibiotic Effect-associated Permeabilization

^c^ NOV, novobiocin; FOS, fosfomycin; RIF, rifampin; ERY, erythromycin; CAZ, ceftazidime; CEF, cephalothin; CIP, ciprofloxacin. MICs (μg/mL**)** are in parenthesis

^d^ Score of Post-Antibiotic Effect-associated Permeabilization. Delay in h caused by exposure to a sub-MIC concentration of an antibiotic added at the indicated time (0 h, 1 h, 2 h) after the beginning of growth in peptide-free medium. S-PAEP was assessed by turdidimetry (Bioscreen)

^e^ Determination was not feasible due to resistance to antibiotic

### Exploitation of PAEP by antibiotics other than novobiocin

To evaluate whether the PAEP induced by P4–9 could be exploited by antibiotics different from novobiocin, we repeated the assays summarized in Fig. 2b using antibiotics from several classes including a macrolide (erythromycin), a first and a third generation cephalosporin (cephalothin and ceftazidime, respectively), a fluoroquinolone (ciprofloxacin) as well as rifampin and fosfomycin. Strain Ps4 is naturally resistant to all these compounds, making it possible to detect sensitization mediated by exposure to the peptide. In all cases, antibiotics were added at a given subinhibitory concentration that in preliminary experiments showed no inhibitory effect on the growth of an untreated Ps4 culture.

**Fig. 3:**
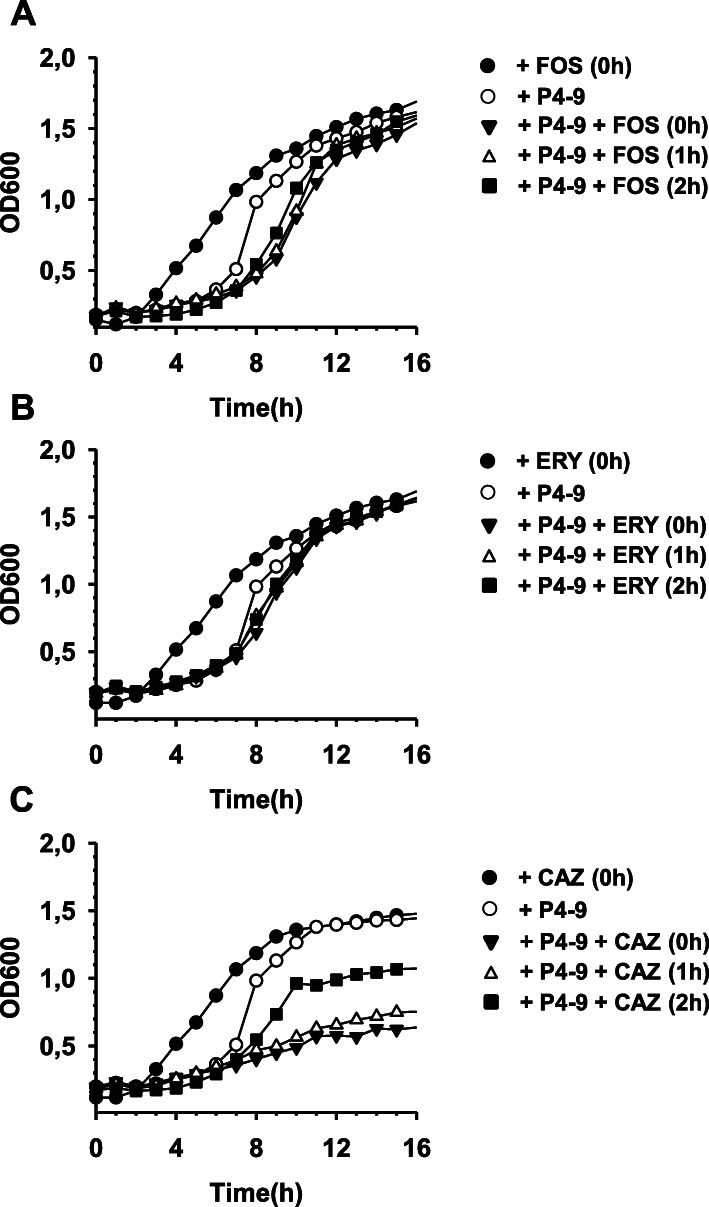
Exploitation of PAEP by sub-MIC concentrations of antibiotics of different classes. Experiments were performed with Ps4 exactly as indicated in Fig. 2b using fosfomycin (**a**), erythromycin (**b**), or ceftazidime (**c**) instead of novobiocin. Fosfomycin, erythromycin and ceftazidime were added at 1/8, 1/8 and 1/4 x MIC, respectively. As control, a P4–9 treated culture was left unexposed to the corresponding antibiotic (open circles). PAEP was calculated as the average of two independent experiments performed in triplicate. Data were analysed by Kruskal–Wallis test followed by Mann–Whitney’s U test and revealed that S-PAEPs induced by fosfomycin and erythromycin were lower than that caused by an equivalent dose of novobiocin (*p* < 0.05; *). For the sake of clarity, error bars are not shown in the figure, although they barely surpass symbol length in most of the cases. For a detailed explanation on PAEP and S-PAEP calculation, see the Methods section

As shown in Fig. 3 and Table 2, overall, treatment with P4–9 sensitized Ps4 to all the antibiotics tested but the magnitude of this effect varied widely from compound to compound. Both, fosfomycin (Fig. 3a) and erythromycin (Fig. 3b) could exploit PAEP but their S-PAEPs were lower than those induced by an equivalent dose of novobiocin (Table 2). Cephalothin, rifampin and ciprofloxacin were the antibiotics causing shortest delays (S-PAEPs ranging from 0.6 to 0.8 h). In marked contrast, ceftazidime outperformed novobiocin and produced S-PAEPs exceeding 14 h (Fig. 3c and Table 2).

### Mechanism of antibiotic resistance expressed by Ps4

The remarkable sensitivity of Ps4 to sub-MIC concentrations of ceftazidime suggested that the mechanisms of resistance to cephalosporins of this strain had been neutralized to a great extent and were hardly functional during the PAEP period. Interestingly, our data indicate that Ps4 possesses two of the main mechanisms of cephalosporin resistance. On the one hand, qPCR analysis revealed that this clinical strain overproduces both the cephalosporinase AmpC and the efflux pump MexAB-OprM, which can hydrolyze ceftazidime or pump the antibiotic out of the cell, respectively [14]. On the other hand, the characterization of the pattern of expression of the cephalosporinase in the presence of an AmpC inhibitor (cloxacillin) showed that Ps4 constitutively overexpresses this β-lactamase, unlike wild type (Fig. S1; note failure to respond to CLO addition in PAO1 compared to Ps4).

### Susceptibility of genetically defined mutants to sensitization during PAEP period

Since Ps4 is a MDR resistant clinical isolate, it probably possesses other mechanisms of antibiotic resistance apart from those mentioned in the previous section. To study the contribution of specific mechanisms of resistance to the degree of susceptibility during the PAEP period, we used genetically defined strains expressing only one of those mechanisms. First, we compared the sensitivity to ceftazidime of Ps4 with that of PAΔADΔDh2Dh3, a strain carrying three mutations in AmpC regulatory genes resulting in the overexpression of a fully induced cephalosporinase ([29]; Fig. S1). Although ceftazidime also sensitized the triple mutant during the PAEP period causing delays of 4.3 h to 3.3 h (Table 2 and Fig. S2A), this strain was more resistant than Ps4 to the cephalosporin. Duplicate assays were performed with PAO1, the parent strain of PAΔADΔDh2Dh3, but the high sensitivity to ceftazidime of the wild type prevented a reliable characterization of PAEP.

We also investigated the behavior of *P. aeruginosa* PAOLC1–6, an isogenic derivative of *P. aeruginosa* PAO1 carrying a mutation that results in constitutive overexpression of MexAB-OprM. Due to the sensitivity of this strain to ceftazidime, instead of this antibiotic we used novobiocin, another specific substrate of MexAB-OprM. As shown in Fig. S2, the mutant (panel C) was almost as sensitive to novobiocin as the wild type (panel B) and values of S-PAEP were comparable in both strains (Table 2).

### PAEP-inducing activity of agents different from P4–9

To study how widespread the ability to induce PAEP was among antimicrobial compounds, we repeated the assays summarized in Fig. 2b using several unrelated antibiotics (gentamicin, ciprofloxacin, imipenem) and one antimicrobial peptide (polymyxin B). As in previous experiments, Ps4 cells were treated with a concentration twice the corresponding MIC of the agent, which in all cases was between 1 and 2 μg/mL (Table 3). After removal of the antimicrobial agent, cells were exposed to sub-inhibitory concentrations of novobiocin (1/8 MIC) that in preliminary experiments had no inhibitory effect on Ps4.

**Table 3:** Post-Antibiotic Effect (PAE) and Score of Post-Antibiotic Effect-associated Permeabilization (S-PAEP) induced by antibiotics on Ps4

Agent used as pre-treatment^**a**^	MIC/MBC (μg/mL)	Final concentration (μg/mL)	PAE^**b**^ (h)	PAEP^**c**^ (h)	S_**nov**_-PAEP^**d**^ (h)		
					0	1	2
GEN	2/2	4	0.75 + 0,01	0	< 0.25	< 0.25	< 0.25
CIP	2/2	4	1.5 + 0,06	0	< 0.25	< 0.25	< 0.25
IPM	2/2	4	0	0	< 0.25	< 0.25	< 0.25
PMB	1/1	2	0.1 + 0,02	2–3	3.2 + 0.06	2.7 + 0.05	1.1 + 0.03

^a^ GEN, gentamicin; CIP, ciprofloxacin; IPM, imipenem; PMB, polymyxin B

^b^ Post-antibiotic effect assessed by turdidimetry (Bioscreen)

^c^ Duration of Post-Antibiotic Effect-associated Permeabilization. PAEs y S-PAEPs values are the average of two independent experiments performed in triplicate. Differences were analyzed by Kruskal–Wallis test followed by Mann–Whitney’s U test

^d^ Score of Post-Antibiotic Effect-associated Permeabilization. Delay in h caused by exposure to a sub-MIC concentration of novobiocin (1/8 x MIC) added at the indicated time (0 h, 1 h, 2 h) after the beginning of growth in peptide-free medium. S-PAEP was assessed by turdidimetry (Bioscreen)

Both gentamicin and ciprofloxacin induced significant PAE on *P. aeruginosa* with the latter antibiotic being twice as potent as the former in this respect (1.5 h vs. 0.75 h; Table 3). In contrast, PAE induced by PMB was negligible (0.1 h), whereas such effect was undetectable in the case of imipenem. Notably, PAE induced by either gentamicin or ciprofloxacin was not associated with permeabilization (i.e. PAEP was not detected). As exemplified by PMB, a poor PAE did not necessarily correlate with a weak PAEP, because this lipopeptide induced PAEP lasting between 2 and 3 h (Table 3), as previously shown for P4–9 (Table 2). In addition, S_NOV_-PAEPs generated by PMB on Ps4 were similar to those measured in P4–9 treated cells exposed to an equivalent concentration of novobiocin (Tables 3 and 2, respectively). Finally, similar to previous observations with P4–9, cells treated with PMB had gradually shorter S-PAEPs the later the novobiocin was added with respect to peptide removal and growth resumption.

### Measuring cell permeabilization with a fluorescent dye (NPN)

**Fig. 4:**
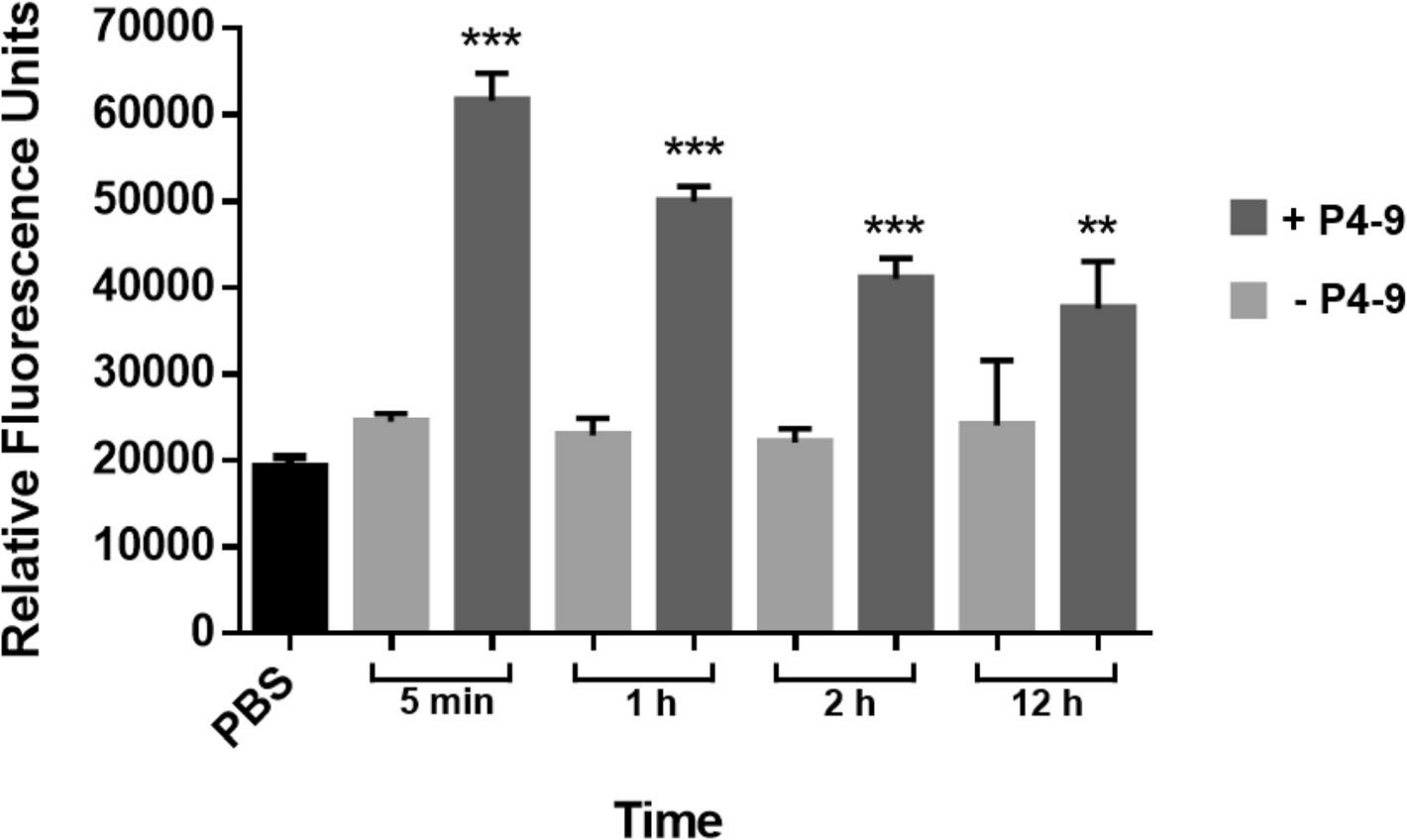
Effect of P4–9 treatment on the permeability of Ps4 cells to N-Phenyl-1-naphthylamine (NPN). Identical suspensions approximately containing 10^7^ fresh mid-log phase CFU/mL were exposed to P4–9 at twice the peptide MIC for 1 h at 37 °C. Peptide was removed by 3 consecutive washes and cells were allowed to grow in fresh MH medium. At the indicated time points, cells were harvested by centrifugation, suspended at an OD600 of 0.6 in PBS, pH 7.2, and added to the wells of a 96-well black polystyrene flat bottom microplate. Then, NPN was dispensed into each well at a final concentration of 100 μM and fluorescence was recorded in a fluorimeter. For each P4–9 treated suspension, an identical untreated suspension was grown in parallel and subjected to an identical procedure. Results shown are the means ± standard deviation of three independent experiments performed in quadruplicate. Data were analyzed using two-sample t-test and statistical differences were extremely significant (***p < 0.001) or very significant. (**p < 0.01). PBS: vehicle (i.e. cell-free) solution containing 100 μM of NPN

Our previous observations suggested that Ps4 envelope had transiently lost its ability to act as a permeability barrier after treatment with P4–9. To confirm this hypothesis we exposed Ps4 to N-Phenyl-1-naphthylamine (NPN), a hydrophobic probe that can only penetrate permeabilized cells. When this occurs, NPN greatly increases its fluorescence. In the assay shown in Fig. 4, cells were treated with P4–9 and after peptide removal by extensive wash, they were allowed to resume growth and were exposed to NPN at different times points. In parallel, untreated suspensions were subjected to identical procedures. When peptide treated cells that had been growing in culture medium for 5 min were exposed to NPN, they reached a level of fluorescence 3 times higher than that measured in untreated cells. In agreement with previous observations, permeability of cells to NPN diminished proportionally to the time of growth after peptide removal. However, compared to untreated cells, P4–9 treated cells continued to take up significantly higher amounts of NPN even after 12 h of growth in peptide-free medium.

### Uptake of fluorescent probes by Ps4 after 20 h of exposure to P4–9

**Fig. 5:**
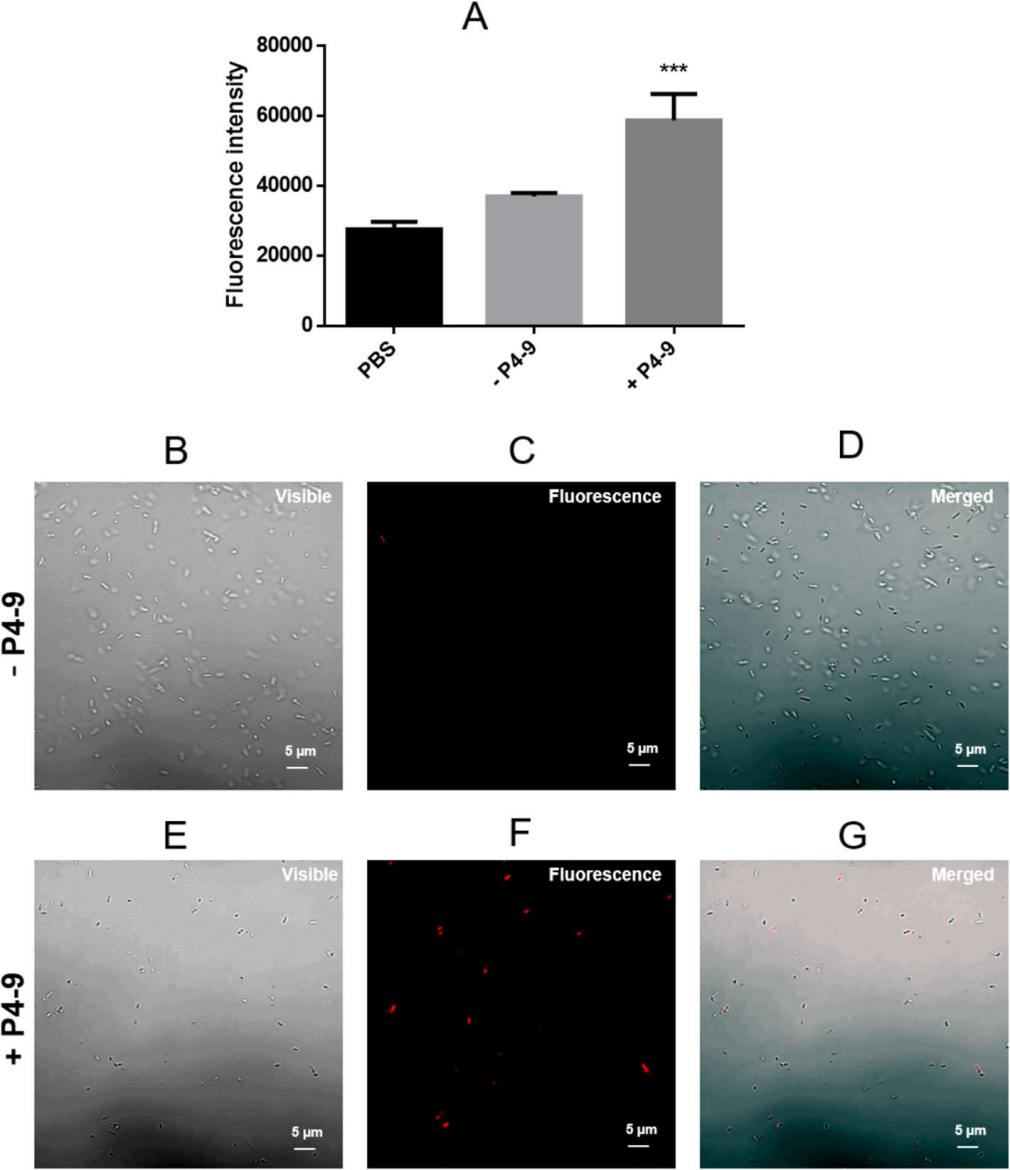
Uptake of fluorescent probes by PS4 cells after 20 h of exposure to P4–9. Cells were treated with P4–9 as described in Fig. 4, and after the washing steps, they were allowed to grow in fresh MH medium without the peptide for 20 h at 37 °C. Then, cells were harvested by centrifugation, washed with PBS and suspended in the same buffer. Partition of NPN (**a**) or propidium iodide (**b**, **c**, **d**, **e**, **f**, **g**) into this cell suspension, labeled as +P4–9 in the figure, was analyzed by fluorimetry and confocal microscopy, respectively. In parallel, duplicate suspensions not treated with P4–9 (labeled as -P4–9 in the figure) were subjected to the same procedure. To record fluorescence emitted by PI (**c**, **f**), suspensions were exposed to an excitation wavelength of 488 nm and images were digitally merged (**d**, **g**) with those of the same field obtained using visible light (**b**, **e**). Results in A are the means ± standard deviation of three independent experiments performed in triplicate. Data were analyzed using two-sample t-test and statistical differences were extremely significant (***p < 0.001). Images shown in lower panels are representative of those visualized in three independent experiments. Percentage of PI-positive cells in 5D and 5G was 2% + 1.52 and 19% + 2.06, respectively. Data (i.e. three independent fields from each experiment) were analyzed using a two-sample t-test and statistical differences were extremely significant (***p < 0.001)

The observation that an antimicrobial peptide could cause such a persistent permeabilization was unexpected and has no precedent in the scientific literature. To confirm these data we used an unrelated technique, confocal microscopy, to analyze the permeability of Ps4 cells that had been growing in MH medium for 20 h after peptide removal (i.e. up to 10^9^ CFU/mL of cell density; see Fig. 1). As fluorochrome, we used propidium iodide (PI), a probe that can only penetrate into permeabilized cells. In parallel, we repeated the fluorimetric NPN test using a suspension identical to that used for the confocal microscopy analysis. Interestingly, the vast majority (98% + 1.52) of cells from the untreated control were able to exclude the dye (Fig. 5d), thus indicating that the culture had not reached the death phase yet. In contrast, a significant number of cells (19% + 2.06; *p* < 0.001) that had been pretreated with P4–9 took up PI and stained red (Fig. 5g), even though they had been growing in the absence of the peptide for 20 h. This is equivalent to an increase of 5 logs in cell size (Fig. 1). Consistent with this observation, P4–9 treated cells grown for 20 h in peptide-free medium showed a highly significant level of fluorescence when exposed to NPN, compared to untreated cells (Fig. 5a).

### Uptake of fluorescent dye by *P. aeruginosa* cells during PAEP period

We ruled out that PI positive cells found in the previous experiment could be dead bacteria killed by the peptide 20 h before because those cells would have been outnumbered by a factor of at least 10^5^ with respect to cells surviving P4–9 treatment (see Fig. 1). Alternatively, we postulated that those red cells could be a subpopulation of individuals arising from progenitors that were permeabilized but not killed by P4–9. If this is correct, the hypothetical permeabilized subpopulation should be detectable shortly after peptide treatment. Thus, we searched for PI positive cells 1 h after peptide treatment by fluorescence microscopy using a PAO1 strain constitutively producing the green fluorescent protein (GFP). In duplicate assays, cells were treated with polymyxin B (PMB), an antimicrobial peptide well known by its envelope disrupting activity.

As shown in Fig. S3, whereas the majority of untreated cells emitted green fluorescence, virtually all cells exposed to PMB took up the dye and stained red (Figs. S3C and S3D). In contrast, cells treated with P4–9 displayed a very heterogeneous staining with colors ranging from green to yellow and –less frequently – red (Fig. Figs. S3E and S3F). A viable count test performed in parallel with this microscopic assay revealed that, even though P4–9 was less bactericidal than PMB at twice its MIC, the treatment with the peptide caused a remarkable mortality in the Ps4 population (2.5 decimal logarithms, approximately) after 60 min. Incubation of P4–9 treated cells with a subinhibitory concentration (1/16 MIC) of novobiocin caused a 3-fold increase in the number of red cells compared to a non-novobiocin treated control (data not shown).

Finally, to investigate in a more direct way the viability of cells taking up PI 20 h after peptide removal, we characterized their motility by confocal microscopy and recorded the movie shown in Fig. S4. This experiment revealed that PI positive cells displayed flagellar motility and were as active as their non-stained counterparts.

### Imaging the surface of P4–9 treated cells by atomic force microscopy

**Fig. 6:**
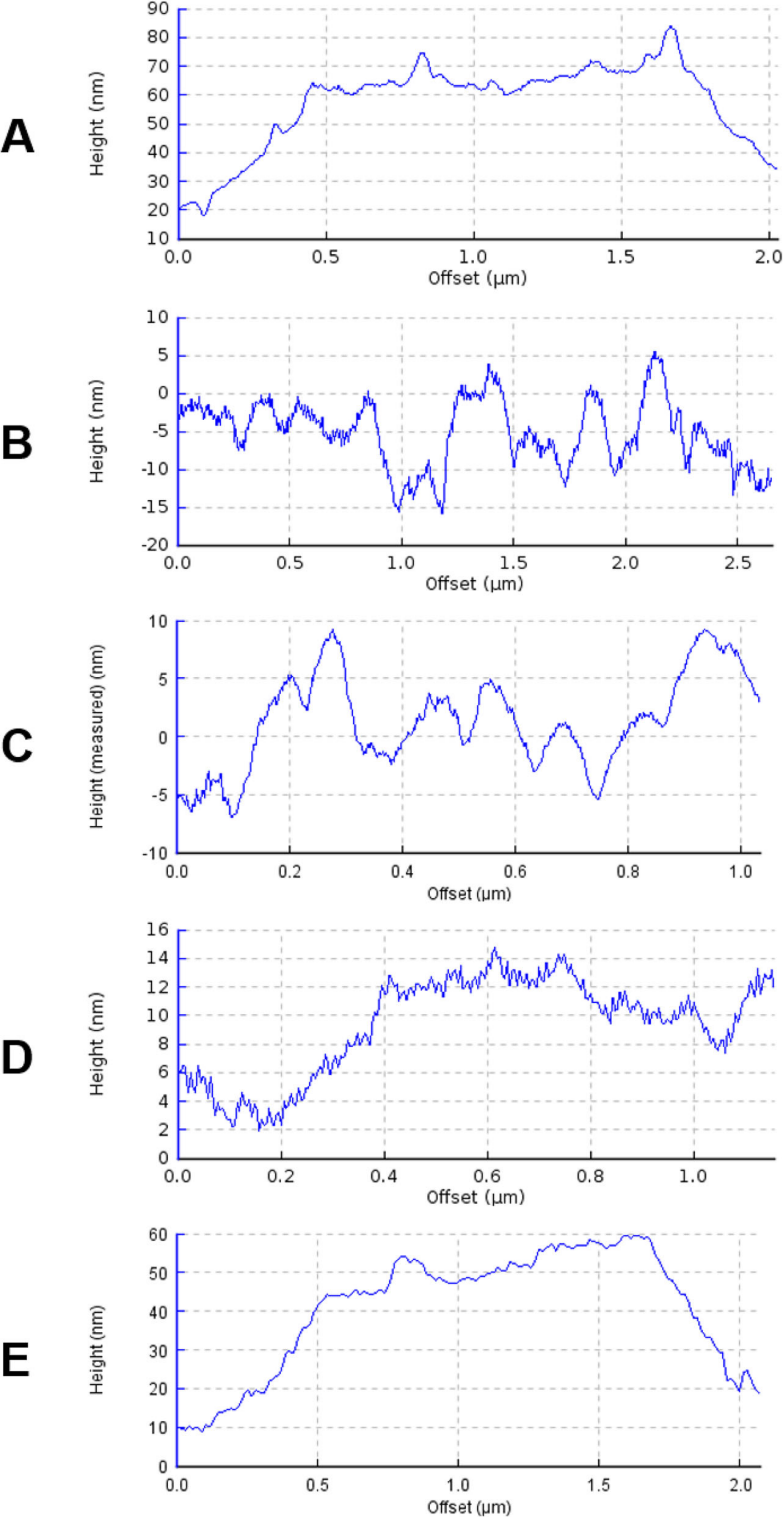
Atomic Force Microscopy images of the surface topography of *P. aeruginosa* cells adsorbed onto glass. Ps4 cells were incubated in MH medium without (**a**) or with (**b**-**e**) antimicrobial agent at twice the MIC for 1 h and visualized either immediately after removal of the agent (**b**; PMB treated control; **c**, P4–9 treated cell) or after 1 h (**d**) or 2 h (**e**) of growth at 37 °C in fresh medium free from antimicrobial agent. Cells selected for the analysis had features representative of those observed in 3 different low magnification fields (*n* = 10 to 20 cells)

To try to visualize potential alterations produced by P4–9 on Ps4, cells were processed as in the previous experiment and their surface was characterized by atomic force microscopy (AFM). In control experiments, PMB was used instead of P4–9. When imaged at low magnification, untreated cells appeared homogeneous and their surfaces looked bright and clear (data not shown). Representative cross sections and two dimensional images of these cells taken at higher magnification revealed a smooth envelope without major grooves or pores (Fig. 6a and Fig. S5A, respectively). In contrast, the equivalent images taken on PMB treated cultures showed abundant dark areas (Fig. S5B) mapping to profound surface depressions in the cross sectional views (Fig. 6b).

On the other hand, cells exposed to P4–9 resembled more their PMB-treated counterparts than the non-treated controls (Fig. S5C). Cross sections confirmed the presence of a rough and jagged surface in P4–9 treated cells but the grooves were less deep and sharp than those caused by PMB treatment (Fig. 6c). Surface alterations were linked to a severe cell flattening both in PMB and P4–9 treated cells (see Y-axis values in Fig. 6b and c). Interestingly, when P4–9 treated cells were allowed to grow in the absence of peptide for 1 h, their surface became much smoother with the only detectable irregularity being evenly spaced tooth-like protrusions of 1 nm of depth, approximately (Fig. 6d). Finally, the overall aspect of P4–9 treated cells that were grown for 2 h in peptide-free medium was very similar to that of untreated cells (Fig. S5D) and their surface appeared regular and smooth in cross sectional views (Fig. 6e).

## Discussion

The ability of certain antimicrobial peptides and membrane-disturbing agents to sensitize bacteria to antibiotics is well known since a long time ago [38–41]. Potentiation of antibiotic activity has been shown to occur both at sub- and suprainhibitory concentrations of the permeabilizing agent [8, 42–44]. However, the present study shows for the first time that such sensitization can persist for several hours after removal of the permeabilizer and is still exploitable for antibiotic enhancement if the organism is allowed to grow for at least 2 h in the absence of the sensitizing agent. This observation may have important therapeutic implications if an agent endowed with PAEP causing activity is intended to be co-administered with antibiotics. Thus, theoretically, the molecule used as antibiotic enhancer would not need to be present to ensure the effectiveness of the treatment, as long as the target organism retains sensitization to the antibiotic. This is especially relevant for antimicrobial peptides which frequently have biological half-lives significantly lower than those of conventional antibiotics [45, 46].

Probably, the so called “post-antibiotic sub-MIC effect” (PA-SME) is the phenomenon most closely related to PAEP. In contrast to PAEP, which involves two different agents, PA-SME results in sensitization to an antibiotic at subinhibitory concentrations after exposure to the same compound at concentrations above its MIC [47–52]. Similarly, increased bacterial susceptibility to phagocytosis or intracellular killing by leukocytes during the PAE phase was described by McDonald and collaborators, which defined this phenomenon as Post-antibiotic Leukocyte Enhancement (PALE) [53]. Finally, other authors measured the PAE induced by combining two or more antibiotics at the same time and reported an enhancement with respect to the PAE of each agent by itself [18].

Taken together, our data indicate that PAEP is the consequence of the severe alterations produced by the peptide in the bacterial envelope and that this effect persists coinciding with the time necessary for the cell to repair its structural damage. Consistent with this, numerous studies demonstrated the existence of morphological and ultrastructural alterations in microorganisms treated with PAE-inducing agents and have postulated that PAE is consequence of such damage [54–60]. In particular, Gottfredsson and collaborators using electron microscopy reported ultrastructural alterations in *Staphylococcus aureus* and *Pseudomonas aeruginosa* which persisted for the duration of the PAE [59]. The hypothesis of recovery from non-lethal damage has also been favored by authors investigating the PAE of antimicrobial peptides such as lactoferricin [26]. The ability of our lead compound P4–9 to induce PAE, as opposed to P4–8 and P4–18, could be due to its structural characteristics including a higher positive net charge and a hydrophobic domain more extensive than that of their counterparts. These properties have been reported to enable antimicrobial agents to incorporate into bacterial membranes and to interfere with cell permeability [61–64].

Consistent with our hypothesis on the mechanism of PAEP, we showed that the duration of this phenomenon (always less than 3 h under our experimental conditions) is a consequence of the peptide treatment and does not vary depending on the type of antibiotic used in the assay. On the contrary, antimicrobials added during the PAEP phase acted with varying efficacy and caused different growth delays, which we designated as S-PAEPs. As we showed, the latter parameter depends not only on the type of antimicrobial agent used but also on the mechanisms of antibiotic resistance expressed by the test organism. Besides, the observation that S-PAEP values progressively diminish as the interval between peptide removal and antibiotic addition increases is in good correlation with our hypothesis. In fact, this would be the expected behavior of a culture undergoing a process of gradual repair of the cell envelope, as we have postulated.

Moreover, the experiments showing co-localization of the fluorescent probes GFP and PI clearly indicate that treatment with P4–9 permeabilizes the envelope of *P. aeruginosa* without causing a total loss of viability. This observation was not unexpected since the concentration of P4–9 used in these tests (64 μg/mL) was still far from the peptide’s lethal concentration (MBC 128 μg/mL). However, we showed that PAEP is not restricted to bacteriostatic agents, as exemplified by PMB, a compound with potent bactericidal activity at its MIC. The ability of PMB to cause PAEP is in all likelihood due to the existence of a small subpopulation of damaged cells surviving PMB exposure, as our microscopic analysis suggests (see white arrows in Fig. S3D). Subsequent growth of these cells after PMB removal would allow detection of PAEP. In fact, in those assays, we confirmed that loss of viability upon PMB exposure was not complete and that such subpopulation of viable cells indeed existed (data not shown).

On the other hand, our results show that PAE and PAEP are not necessarily associated. Thus, PAEP was not detectable in cells treated with compounds causing prominent PAEs, such as ciprofloxacin or gentamicin. Conversely, the antimicrobial peptide producing the lowest PAE (PMB; PAE = 0.1 h) caused a potent PAEP persisting for at least 2 h. Although it is necessary to study whether other membrane disturbing agents can induce PAEP, our data suggest that this phenomenon may be restricted to those antimicrobials causing damage to the bacterial envelope, a hallmark of PMB and other antimicrobial peptides [65, 66]. In contrast with our observations, focused on inhibitory effects, the bactericidal activity of some antibiotics was reported to significantly decrease during the PAE phase induced by a different antimicrobial [67, 68]. These studies concluded that, compared to untreated controls, the time required to kill the microorganism was several hours longer during the PAE period and paralleled the duration of the PAE. However, these studies were not performed using antimicrobial peptides as PAE inducing agents.

AFM analysis confirmed that exposure of *P. aeruginosa* to P4–9 causes superficial alterations similar to -although less pronounced than- those observed in cells treated with PMB. Using this technique, other authors reported similar surface changes in bacteria exposed to antimicrobial peptides [69, 70] including colistin [71] and synthetic peptides derived from bovine lactoferricin [72]. Furthermore, AFM assays show that, when allowed to grow in the absence of peptide, P4–9 treated cells undergo a progressive process of recovery that appears to be complete after 2 h of growth resumption. However, even after this period of time, cells retain sensitivity to antibiotics, as demonstrated in PAEP assays, and require additional time (consistently less than an hour) to fully recover their ability to exclude antibiotics. This observation suggests that the indirect technique based on susceptibility to antibiotics is more sensitive than a direct imaging-based technique such as AFM to detect PAE-associated alterations.

Finally, the present work shows for the first time that the descendants of bacteria surviving exposure to a membrane disturbing agent continue to be permeable to NPN and PI even after 20 h of growth post-exposure. While the molecular basis of such long-term permeability is presently unknown, our results show that the hypothetical alterations responsible for this phenomenon do not confer antibiotic sensitivity and are undetectable by AFM.

In the current context of severe limitation of therapeutic options against MDR pathogens, the antibiotic sensitization phenomenon identified in this study might be clinically relevant. It is worth to note that P4–9 did not act in synergy with novobiocin on *P. aeruginosa* at sub-inhibitory concentration (FIC index > 0.5; data not shown). However, cells treated with the peptide above its MIC became temporarily sensitive (i.e. for at least 2 h), not only to novobiocin but also to a wide variety of clinically used antibiotics such as cephalothin, fosfomycin, erythromycin, rifampin, ceftazidime, ciprofloxacin, etc. Also, peptide pre-treatment efficiently neutralized to a great extent two major mechanisms expressed by a MDR *P. aeruginosa* strain, namely the efflux pump system MexAB-OprM and the AmpC cephalosporinase. Nevertheless, these in vitro observations should be first validated in animal models of infection as a first step in the evaluation of the therapeutic utility of PAEP.

## Conclusions

We showed for the first time that a multidrug-resistant strain of *P. aeruginosa* can be rendered susceptible for several hours to subinhibitory concentrations of antibiotics upon treatment with an antimicrobial peptide. Descendants of bacteria surviving exposure to the peptide retain a significant level of permeability to hydrophobic compounds after 20 h of growth in the absence of the peptide. We designated this persistent sensitization to antibiotics occurring in the absence of the sensitizing agent as Post-Antibiotic Effect associated Permeabilization (PAEP). This phenomenon may have important therapeutic implications for a combined peptide-antibiotic treatment because the peptide would not need to be present to exert its antibiotic enhancing activity as long as the target organism retains sensitization to the antibiotic.

## Supplementary information

**Additional file 1: Table S1.** Antibiogram of *Pseudomonas aeruginosa* 4158–02 Ps4. The qualitative pattern of antibiotic susceptibility (antibiogram) of the clinical isolate *P. aeruginosa* Ps4 was obtained using an automated Vitek II system (bioMérieux) equipped with the AST-NO22 card. Results were interpreted according to European Committee on Antimicrobial Susceptibility Testing (EUCAST) breakpoints. **Fig. S1.** Phenotypic characterization of AmpC betalactamase production in *P. aeruginosa* strains used in this study. Suspensions of the indicated strains adjusted to a turbidity of 0.5 McFarland were inoculated onto Mueller Hinton (MH) plates without (left panels) or with the AmpC betalactamase inhibitor cloxacillin (right panels; CLO) and grown for 24 h at 37 °C. Note the increase in the zone of growth inhibition in the two lower rows compared to two the upper rows as a result of CLO addition. See relevant features of these strains in Table 1. Assays were performed according to CLSI guidelines [73] and following the method of De Champs (De Champs et al. 2002 [74]). AmpC inhibition was considered significant when the ceftazidime zone diameter increased by > 10 mm. **Fig. S2.** Susceptibility of genetically defined mutants to sensitization during PAEP period. Experiments were performed exactly as indicated in Fig. 2b using the following strains and antibiotics. (A), PAΔADΔDh2Dh3 (AmpC overexpressing) strain treated with 1/4 x MIC of ceftazidime; (B), wild type PAO1 or (C), PAOLC1–6 treated with 1/16 x MIC of novobiocin. Results shown are the average of two independent experiments performed in triplicate. Data were analysed by Kruskal–Wallis test followed by Mann–Whitney’s U test which revealed that the triple mutant was more resistant than Ps4 to the cephalosporin in the first two time-points (0 h, 1 h; *p* < 0.05; *), and that PAOLC1–6 was more resistant to novobiocin than Ps4 only in the second time point (1 h; p < 0.05; *). For the sake of clarity, error bars are not shown in the figure, although they barely surpass symbol length in most of the cases. **Fig. S3.** Uptake of propidium iodide by GFP-expresing *P. aeruginosa* cells during PAEP period. Representative fluorescent microscopy images obtained at 1000x of undiluted samples (left panels) or the corresponding 10-fold dilution (right panels). Cultures were either treated with 2 x MIC of polymyxin B (C, D) or with 2 x MIC of peptide P4–9 (E, F) for 1 h and, after thorough washing, exposed to propidium iodide. As control (A, B), a duplicate culture was left untreated and processed in parallel with the treated cultures. White arrows in panel D point to cells emitting green fluorescence. In each panel, selected images are representative of a total of 10 different fields inspected. **Fig. S4.** Assessment of motility of PS4 cells that were allowed to grow for 20 h after peptide P4–9 removal. A. Cells were treated with P4–9 at twice its MIC for 1 h and, after peptide removal by extensive washing, they were allowed to grow in fresh MH medium for 20 h. B. a duplicate suspension was boiled for 5 min. Then, for imaging, culture samples were stained with propidium iodide and immediately visualized by confocal microscopy at 630x and an excitation wavelength of 488 nm. Images record the behavior of the entire cell population (10 different fields inspected in each panel). Similar results were observed in two other independent experiments. Time elapsed in the recordings was approximately 8 s. **Fig. S5.** Two-dimensional Atomic Force Microscopy imaging of *P. aeruginosa* Ps4. Cells were exposed to 2 x MIC of either polymyxin B (B) or peptide P4–9 (C and D) for 1 h and, after removal of the agent by repeated washing, the surface of representative cells was scanned by AFM immediately after the washes (B and C) or after 2 h (D) of growth at 37 °C in fresh medium free from P4–9. As a control, a duplicate culture was left untreated, washed thoroughly and analyzed by AFM (A). Cells selected for the analysis had features representative of those observed in 3 different low magnification fields (*n* = 10 to 20 cells). (PPTX 55674 kb)

## Data Availability

All data generated and analyzed during this study are included in this published article and its supplementary information files.

## Abbreviations

AFM: atomic force microscopy
AMPs: antimicrobial peptides
CAZ: ceftazidime
CEF: cephalothin
CFU: colony forming unit
CIP: ciprofloxacin
CLO: cloxacillin
CLSI: clinical and laboratory standards institute
ERY: erythromycin
EUCAST: European committee on antimicrobial susceptibility testing
FIC: fractional inhibitory concentration
FITC: fluorescein isothiocyanate
FOS: fosfomycin
FMOC: 9-fluorenylmethyloxycarbonyl
GEN: gentamicin
GFP: green fluorescence protein
IPM: imipenem
MBC: minimal bactericidal concentration
MDR: multi-drug resistant
MH: Mueller Hinton
MIC: minimal inhibitory concentration
NOV: novobiocin
NPN: N-Phenyl-1-naphthylamine
OD: optical density
PAE: post-antibiotic effect
PAEP: post-antibiotic effect associated permeabilization
PA-SME: post-antibiotic sub-MIC effect
PBS: phosphate buffer saline
PI: propidium iodide
PMB: polymyxin B
qPCR: quantitative polymerase chain reaction
RIF: rifampin
RP-HPLC: reversed phase high-performance liquid chromatography
S-PAEP: score of post-antibiotic effect-associated permeabilization
TRITC: tetramethylrhodamine isothiocyanate
TSA: trypticase soy agar
TSB: trypticase soy broth
