# Supplementary material for: A synthetic peptide sensitizes multi-drug resistant Pseudomonas aeruginosa to antibiotics for more than two hours and permeabilizes its envelope for twenty hours
Source: J Biomed Sci. 2020 Aug 6;27:85. doi: 10.1186/s12929-020-00678-3 (PMC7412836; doi:10.1186/s12929-020-00678-3)
Supplement: Supplementary file 1 — Additional file 1: Table S1. Antibiogram of Pseudomonas aeruginosa 4158–02 Ps4. The qualitative pattern of antibiotic susceptibility (antibiogram) of the clinical isolate P. aeruginosa Ps4 was obtained using an automated Vitek II system (bioMérieux) equipped with the AST-NO22 card. Results were interpreted according to European Committee on Antimicrobial Susceptibility Testing (EUCAST) breakpoints. Fig. S1. Phenotypic characterization of AmpC betalactamase production in P. aeruginosa strains used in this study. Suspensions of the indicated strains adjusted to a turbidity of 0.5 McFarland were inoculated onto Mueller Hinton (MH) plates without (left panels) or with the AmpC betalactamase inhibitor cloxacillin (right panels; CLO) and grown for 24 h at 37 °C. Note the increase in the zone of growth inhibition in the two lower rows compared to two the upper rows as a result of CLO addition. See relevant features of these strains in Table 1. Assays were performed according to CLSI guidelines [73] and following the method of De Champs (De Champs et al. 2002 [74]). AmpC inhibition was considered significant when the ceftazidime zone diameter increased by > 10 mm. Fig. S2. Susceptibility of genetically defined mutants to sensitization during PAEP period. Experiments were performed exactly as indicated in Fig. 2b using the following strains and antibiotics. (A), PAΔADΔDh2Dh3 (AmpC overexpressing) strain treated with 1/4 x MIC of ceftazidime; (B), wild type PAO1 or (C), PAOLC1–6 treated with 1/16 x MIC of novobiocin. Results shown are the average of two independent experiments performed in triplicate. Data were analysed by Kruskal–Wallis test followed by Mann–Whitney’s U test which revealed that the triple mutant was more resistant than Ps4 to the cephalosporin in the first two time-points (0 h, 1 h; p < 0.05; *), and that PAOLC1–6 was more resistant to novobiocin than Ps4 only in the second time point (1 h; p < 0.05; *). For the sake of clarity, error bars are not shown in t [file 12929_2020_678_MOESM1_ESM.pptx]

## Slide 1
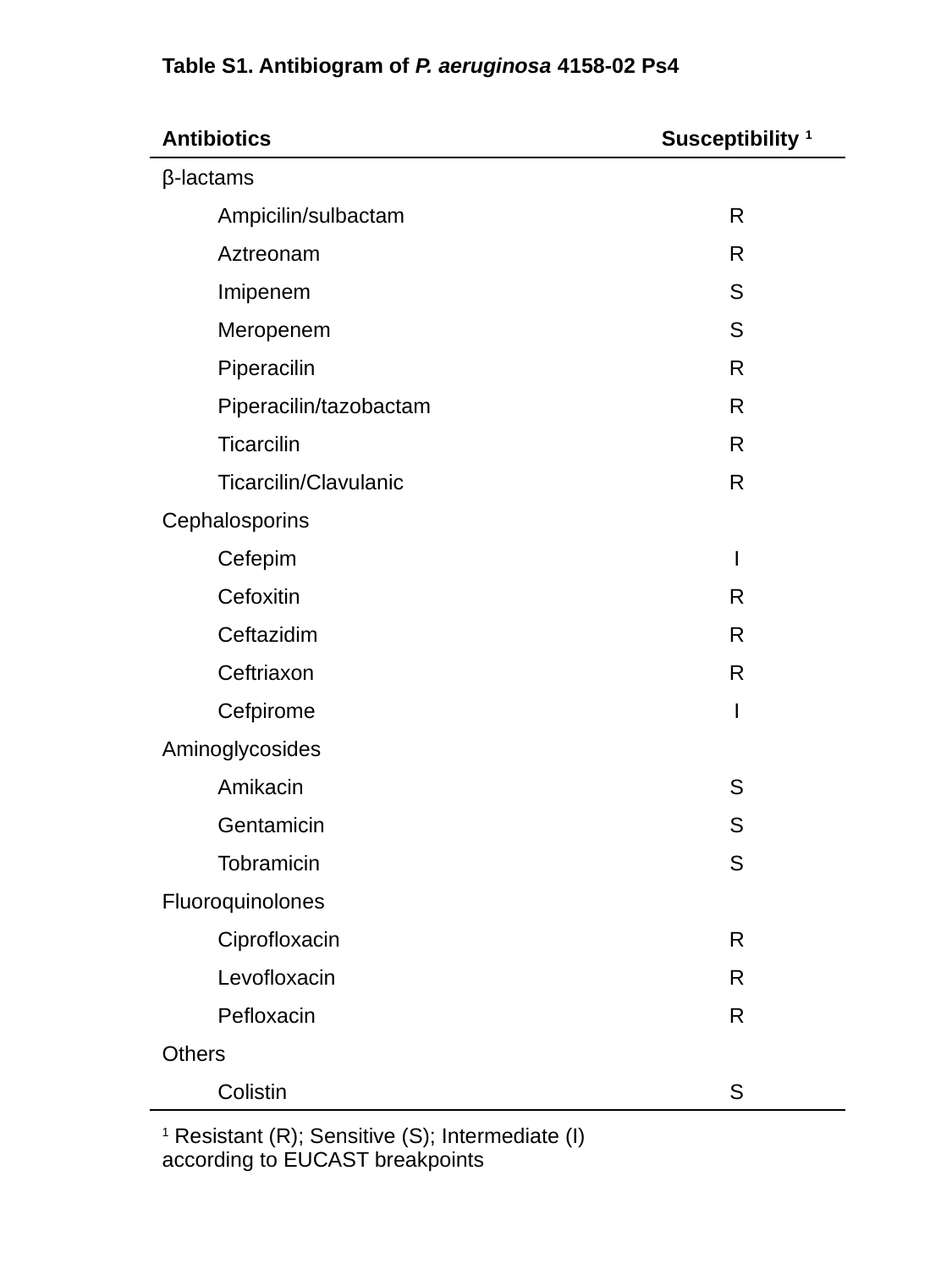

| Table S1. Antibiogram of P. aeruginosa 4158-02 Ps4 | | |
| --- | --- | --- |
| Antibiotics | | Susceptibility 1 |
| β-lactams | | |
| | Ampicilin/sulbactam | R |
| | Aztreonam | R |
| | Imipenem | S |
| | Meropenem | S |
| | Piperacilin | R |
| | Piperacilin/tazobactam | R |
| | Ticarcilin | R |
| | Ticarcilin/Clavulanic | R |
| Cephalosporins | | |
| | Cefepim | I |
| | Cefoxitin | R |
| | Ceftazidim | R |
| | Ceftriaxon | R |
| | Cefpirome | I |
| Aminoglycosides | | |
| | Amikacin | S |
| | Gentamicin | S |
| | Tobramicin | S |
| Fluoroquinolones | | |
| | Ciprofloxacin | R |
| | Levofloxacin | R |
| | Pefloxacin | R |
| Others | | |
| | Colistin | S |
| 1 Resistant (R); Sensitive (S); Intermediate (I) according to EUCAST breakpoints | | |
| | | |

## Slide 2
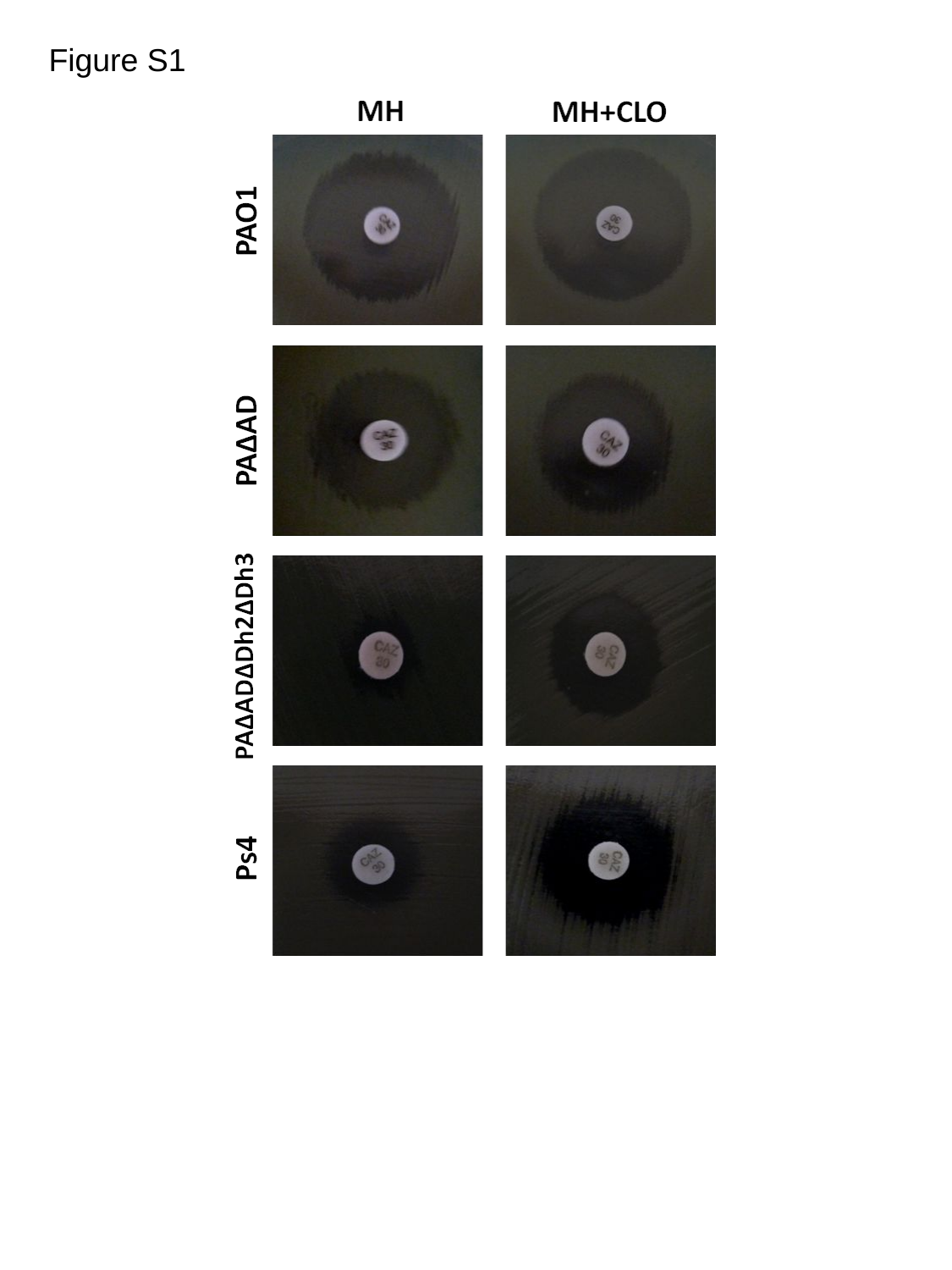

Figure S1

## Slide 3
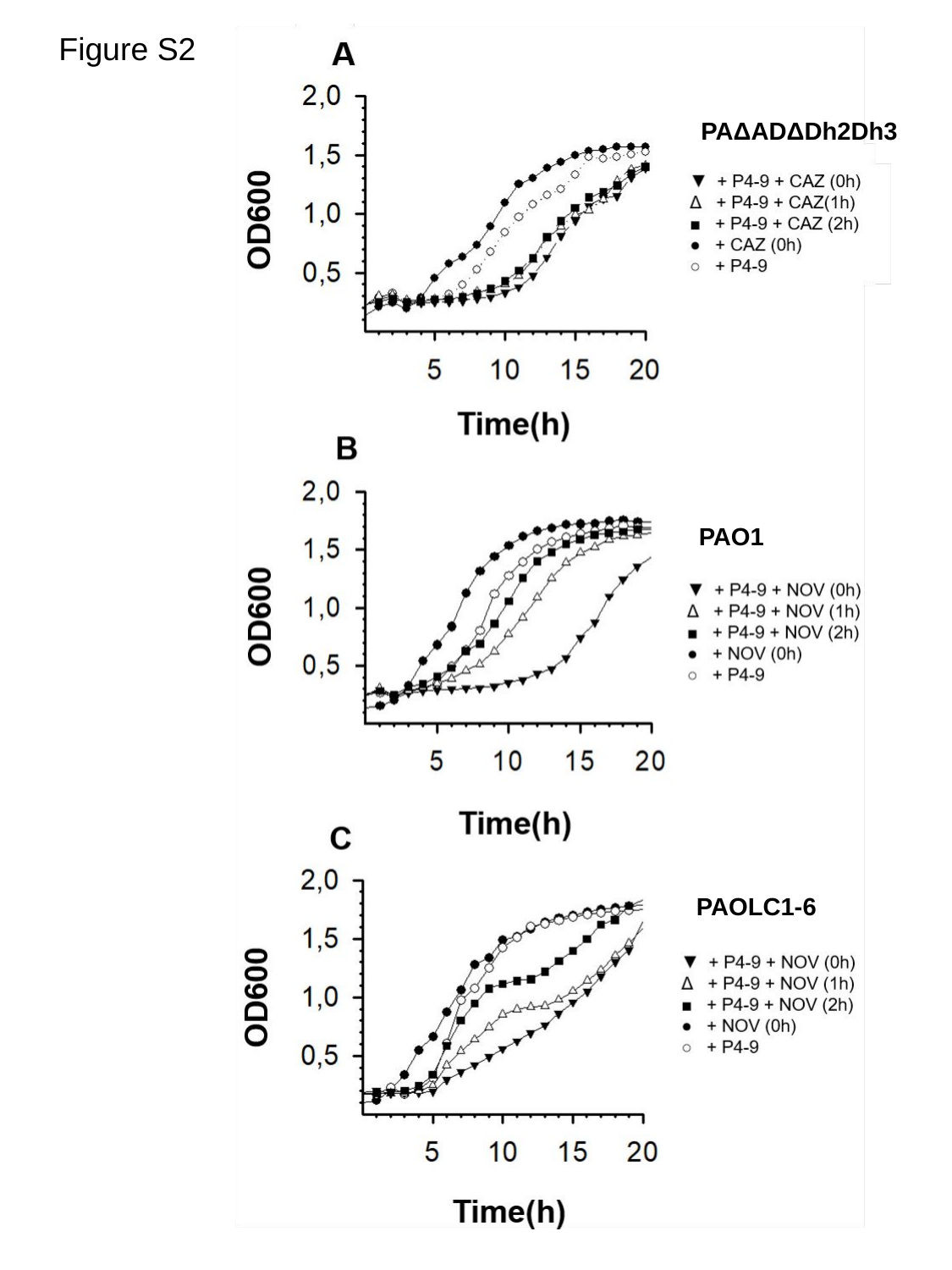

Figure S2
PAΔADΔDh2Dh3
PAO1
PAOLC1-6

## Slide 4
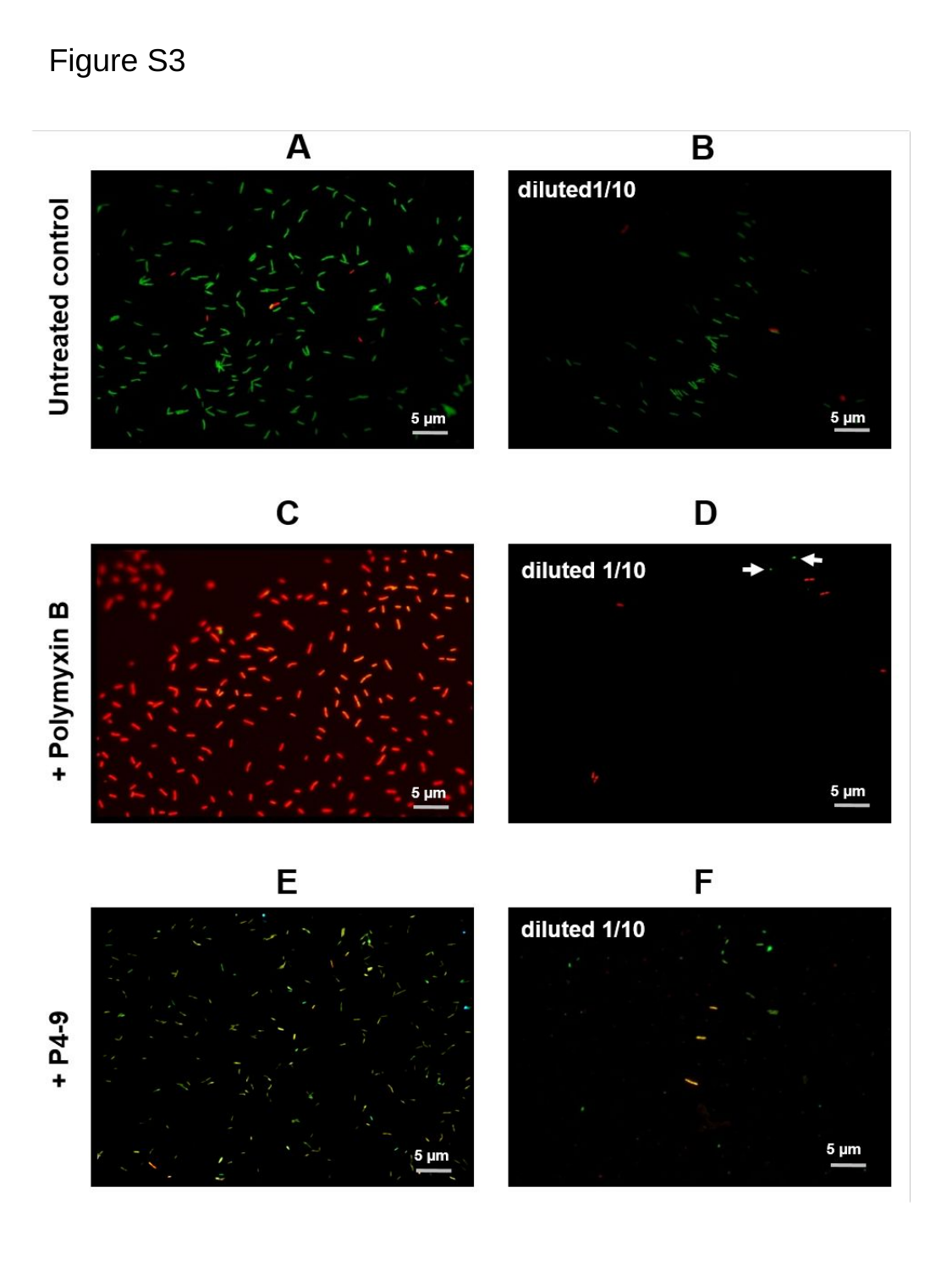

Figure S3

## Slide 5
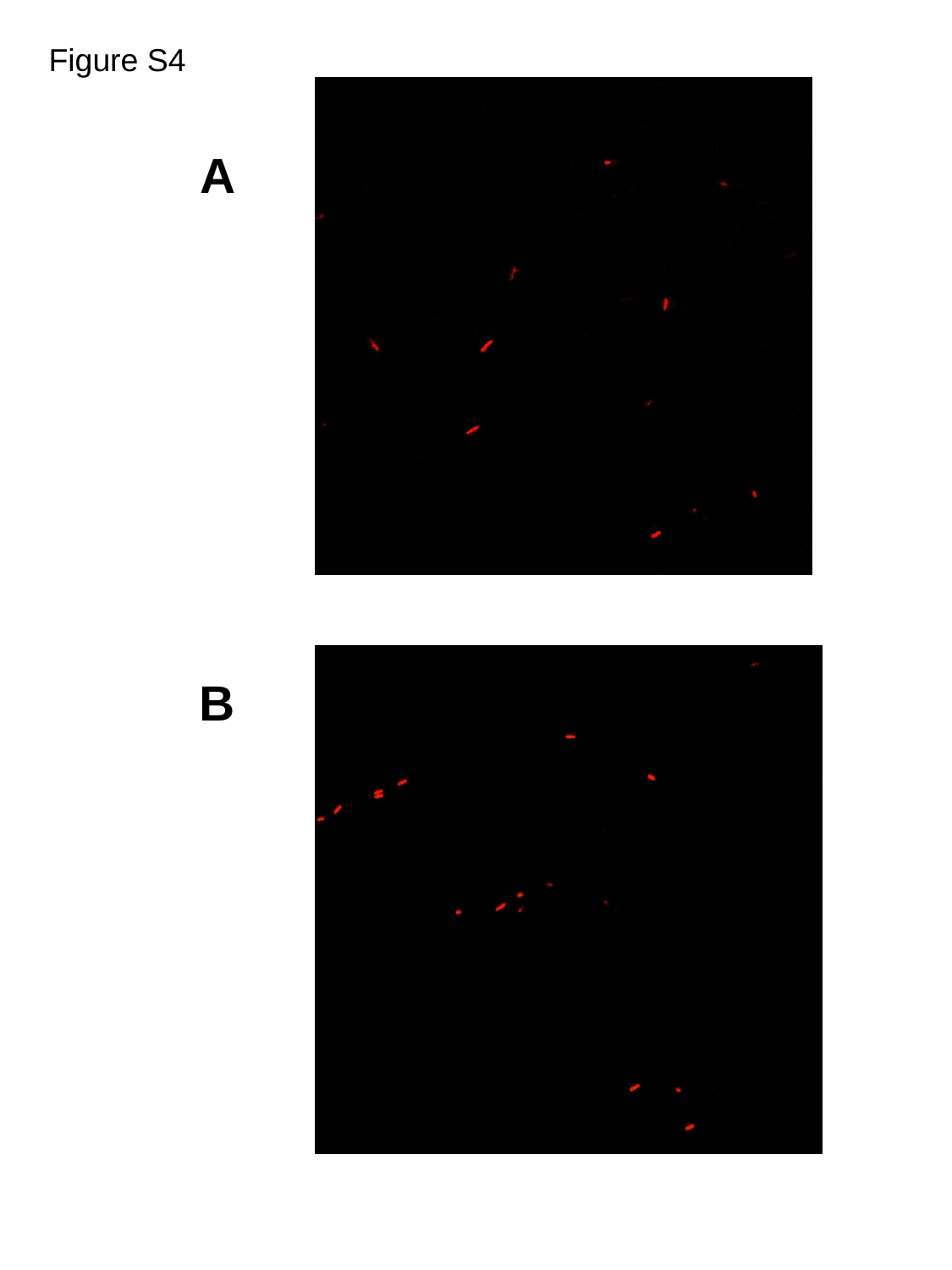

Figure S4
A
B

## Slide 6
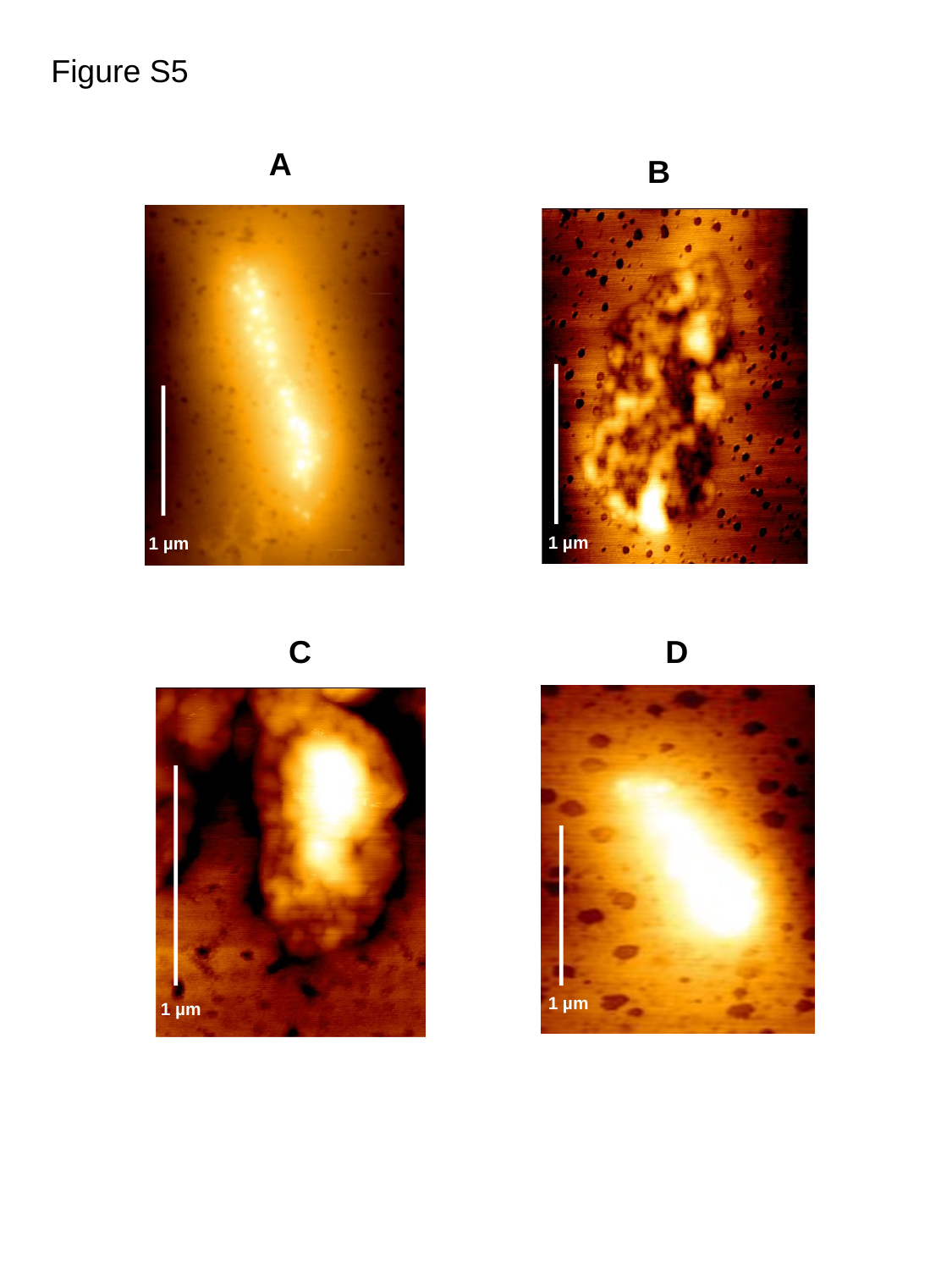

Figure S5
A
B
1 µm
1 µm
C
D
1 µm
1 µm
